# Sarcomas: New Biomarkers and Therapeutic Strategies

**DOI:** 10.3390/cancers13205213

**Published:** 2021-10-18

**Authors:** Tomoki Nakamura

**Affiliations:** Department of Orthopaedic Surgery, Mie University Graduate School of Medicine, Tsu 514-8507, Japan; tomoki66@clin.medic.mie-u.ac.jp

Bone and soft tissue sarcoma (STS) are heterogeneous diseases comprised of various molecular and histologic subtypes [1]. During the 1980s and 1990s, due to advances in chemotherapy and surgical techniques, the survival rate reached approximately 70–80% in patients with sarcoma [2,3,4]. However, survival rates have not improved during the last 30 years. New biomarkers and therapeutic strategies are necessary to further improve the prognosis. The aim of this Special Issue is to investigate new biomarkers and their possible application for the treatment in patients with sarcoma. This series of 12 articles (10 original articles, 2 reviews) is presented by international researchers.

Osteosarcoma is not a cancer clearly characterized by genetic mutations or oncogenic expressions. Therefore, it is remarkably interesting to investigate new prognostic signatures and therapeutic targets. Pandya et al. found that the identification of the over-amplification of the MYC-RAD21 genes associated with patients that have lower overall survival (OS), and the therapeutic approach to use a BET inhibitor and CHK1 inhibitor appears to have significant negative consequences on the osteosarcoma cells.

In addition to osteosarcoma, Ewing’s sarcoma (ES) is also the most frequently observed primary malignant bone tumor in the second decade of life [5]. Mullard et al. demonstrated that GANT61, an inhibitor of the transcriptional factor Gli1, reduced ES primary tumor growth using a preclinical model of ES. In vitro experiments showed that GANT61 decreases the viability of ES cell, mainly through its ability to induce caspase-3/7-dependent cell apoptosis. GANT61 may be a promising therapeutic strategy for inhibiting the progression of primary ES tumors.

There is no single clinical examination to facilitate the accurate diagnosis of STS, which requires comprehensive consideration of several examinations. Fujibuchi et al. demonstrated the relationship between pretreatment clinical parameter and accurate diagnosis of STS. Multivariable analysis revealed that tumor size, white blood cell count, hemoglobin count, C-reactive protein level, and lactate dehydrogenase level were significant predictive factors for sarcoma.

In the patients with STS, surgical resection is the standard treatment for STS. Götzl et al. reported that the retrospective analysis of 290 patient showed significantly clearer margin resections in cases with free-flap reconstructions. The surgical margin is a critical variable for achieving local tumor control.

Adjuvant radiotherapy is also important for achieving local tumor control [6]. Senolytic drugs represent a highly promising field as a new therapy approach to drive senescent cancer cells towards cell death to enhance treatment response. Lafontain et al. demonstrated that the Bcl-2 family of anti-apoptotic proteins in irradiated senescent sarcoma cells represents a senotherapeutic target to improve the cell death response in radiotherapy.

Using a resected tumor sample, Nakamura et al. described that a high expression of both interleukin-6 (IL-6) and IL-6 receptors was a prognostic factor for OS and metastasis-free survival (MFS). They found that this high expression indicated that the patient had a poor prognosis for OS and MFS. IL-6 is an essential cytokine in the regulation of inflammation [7]. The inflammatory microenvironment plays an important role in the development of cancer [8].

The median survival time of patients with advanced STS is typically <12 months [9]. Therefore, the development of new drug should be expected. Su et al. identified that clinically relevant alterations in the tumor immune microenvironment, where intratumoral cell–cell interactions between marker-defined CD11c+ APCs and CD8+ T cells emerged as a novel prognostic marker for improving survival in patients with STS.

Dadone-Montaudié et al. evaluated the therapeutic potential of the pan-FGFR inhibitor erdafitinib to treat dedifferentiated liposarcoma (DDLPS). They detected overexpression of FGFR1 and/or FGFR4 in a well-differentiated/dedifferentiated liposarcoma (WDLPS/DDLPS) and demonstrated correlation of this expression with poor prognosis. Erdafitinib treatment reduced cell viability, inducing apoptosis and strong inhibition of the ERK1/2 pathway.

Miolo et al. tried to identify new serum prognostic biomarkers in STS patients treated with the trabectedin regimen. They showed that citrulline, an amino acid belonging to the arginine metabolism, represents an important metabolic signature that may related with survival in patients with STS.

Recent studies on metastatic colonization have confirmed the hypothesis that certain tissue microenvironments may be preordained to be intrinsically hospitable to disseminated cancer cells [10]. Pouliquen et al. used proteomic quantitative analyses to further demonstrate that liver microenvironment might determine the metastatic potential of the tumor and identified, by sequential subtraction analyses, a few key players in experimental models of peritoneal sarcomatoid malignant mesothelioma.

In addition to original articles, this Special Issue includes two reviews. Fujiwara et al. reviewed role of tumor-associated macrophage in sarcomas. Tumor-associated macrophages (TAMs) are a major component of tumor-infiltrating immune cells in the tumor microenvironment and have a dominant role as orchestrators of tumor-related inflammation. TAM-targeted therapy as a novel treatment approach for sarcomas should be expected. Napolitano et al. reviewed the treatment of desmoid including low-dose chemotherapy and treatment with tyrosine kinase inhibitors.

We expect that these possible biomarkers could be useful for detecting sarcomas earlier and making aggressive treatment strategies to improve survival in patients with sarcomas when those markers are raised.

We also hope that researchers enjoy reading this Special Issue on present and future biomarkers and therapeutic strategies in sarcomas.
